# Polymer-Based Materials Loaded with Curcumin for Wound Healing Applications

**DOI:** 10.3390/polym12102286

**Published:** 2020-10-06

**Authors:** Sibusiso Alven, Xhamla Nqoro, Blessing Atim Aderibigbe

**Affiliations:** Department of Chemistry, University of Fort Hare, Alice Campus, Eastern Cape 5700, South Africa; 201214199@ufh.ac.za (S.A.); 201300305@ufh.ac.za (X.N.)

**Keywords:** wound healing, wound dressings, sponges, curcumin, skin regeneration, sponges, hydrogels, nanofibers

## Abstract

Some of the currently used wound dressings have interesting features such as excellent porosity, good water-absorbing capacity, moderate water vapor transmission rate, high drug loading efficiency, and good capability to provide a moist environment, but they are limited in terms of antimicrobial properties. Their inability to protect the wound from microbial invasion results in wound exposure to microbial infections, resulting in a delayed wound healing process. Furthermore, some wound dressings are loaded with synthetic antibiotics that can cause adverse side effects on the patients. Natural-based compounds exhibit unique features such as good biocompatibility, reduced toxicity, etc. Curcumin, one such natural-based compound, has demonstrated several biological activities such as anticancer, antibacterial and antioxidant properties. Its good antibacterial and antioxidant activity make it beneficial for the treatment of wounds. Several researchers have developed different types of polymer-based wound dressings which were loaded with curcumin. These wound dressings displayed excellent features such as good biocompatibility, induction of skin regeneration, accelerated wound healing processes and excellent antioxidant and antibacterial activity. This review will be focused on the *in vitro* and *in vivo* therapeutic outcomes of wound dressings loaded with curcumin.

## 1. Introduction

Wound healing is dynamic and complex biological process that involves several consecutive healing phases [1]. Good clinical wound management is very important to combat various complications that may occur during the healing process. The management of wounds includes the control of microbial infections, cleaning the wound environment to make it free from foreign substances and the selection of a suitable wound dressing [2,3,4,5]. An ideal wound dressing should have several important features such as high porosity, high water-absorbing capacity, good water vapor transmission rate, non-toxicity, excellent gaseous permeation and ability to maintain a moist environment to speed up the wound healing process [6,7,8,9]. The shortcomings of wound dressings that usually hamper the wound healing process include poor antimicrobial activity, weak antioxidant efficacy, need for frequent changing of wound dressing, poor biodegradability and poor mechanical properties, etc. [10].

Wound dressings can be developed from a combination of bio- and synthetic polymers. Biopolymers suffer from poor mechanical properties that can be overcome by combining them with synthetic polymers [11]. Some of the biopolymers that are utilized in the preparation of wound dressings include hyaluronic acid (HA), alginate [12], dextran, elastin, cellulose [8], chitosan [13], fibrin, chitin, collagen, gelatin, etc. [8,14]. The advantages of biopolymers are their non-toxicity, non-antigenicity, inertness, bioadhesiveness, biocompatibility, biodegradability, good hemostatic effects, and antimicrobial properties. The synthetic polymers used in wound dressing formulations include poly(ethylene glycol) (PEG)/poly(ethylene oxide)(PEO), poly(vinyl pyrrolidone) (PVP), poly(vinyl alcohol) (PVA), [15], poly(hydroxyethyl methacrylate) (PHEMA), polyurethanes (PUs), poly (α-esters) [e.g., poly(lactic-co-glycolic acid) (PLGA), polyglycolic acid (PGA), polylactide (PLA), poly(ε-caprolactone)(PCL)] etc. [16,17,18]. These polymers exhibit good mechanical properties but suffer from poor biocompatibility. They can be combined with biopolymers resulting in hybrid polymers that display good physicochemical properties.

The good features of wound dressings that are designed from the aforementioned polymers can be further enhanced by loading them with natural-based bioactive agents. Curcumin, also called diferuloylmethane, is a natural-based polyphenol and a bioactive agent obtained from the rhizomes of *Curcuma longa* (turmeric) [19,20,21]. *Curcuma longa* contains yellow pigments known as curcuminoids [22,23,24,25]. The biological activity of turmeric is mainly due to curcumin. Curcumin has been broadly employed in the treatment of wounds because of its interesting therapeutic activities such as its anti-inflammatory, antibacterial and anti-oxidant activity [11]. It has attracted the wide attention of many biomedical researchers, not only because of its biological effects, but also because of its excellent biocompatibility and non-toxicity. There are numerous facts that support the status of curcumin as safe and effective [19]. The U.S. Food and Drug Administration has categorized the curcumin molecule as ‘Generally Regarded as Safe’ (GRAS) [20]. However, its biomedical application is limited by its poor water solubility and bioavailability. These limitations can be overcome by loading curcumin into polymer-based materials. This review manuscript reports the *in vitro* and *in vivo* therapeutic outcomes of polymeric wound dressings loaded with curcumin.

## 2. Classification of Wounds and Phases of Wound Healing Process

**Figure 1:**
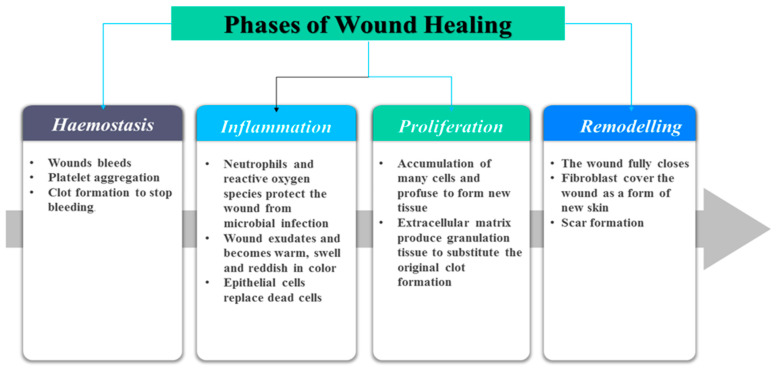
Sequential phases of wound healing process.

A wound is a physical disruption of the skin architecture due to trauma resulting in fluid loss, injury and pain to the individual [26]. Wounds are classified as chronic and acute wounds based on their duration and the nature of the healing process [27]. Chronic wounds are injuries that fail to heal via the normal wound healing process in a timely manner [28]. Some examples of chronic wounds include diabetic wounds, burn wounds, ulcer wounds, etc. [29,30]. Acute wounds are caused by accidental injury or surgical procedure and these wounds heal within the expected period of 8–12 weeks depending on the size and depth of the wound in the skin [31]. The healing process involves four sequential wound healing phases namely: hemostasis, inflammatory, proliferative phase and and remodeling phase (Figure 1) [32,33].

The hemostasis phase occurs immediately after the injury and the exposed sub-endothelium, tissue factor and collagen stimulate platelet aggregation that causes degranulation and releasing of growth factors (GFs) and chemokines to produce a blood clot [34,35]. In the inflammatory phase neutrophils, proteases and reactive oxygen species (ROS) protect the wound from microbial infections and cleanse it from the debris to provide a good environment for an accelerated healing process. The exudate leucocytes are responsible for the redness and sometimes erythema, warmth and swelling of the damaged skin [36]. The epithelial cells invade the wound environment to replace dead cells. The duration of the hemostasis and inflammatory phases depends on the gravity of the injury [37].

In the proliferative phase, there is a proliferation of cells and profuse connective tissue. Extracellular matrix (ECM), including HA, proteoglycans and other species produce a granulation tissue to substitute the original clot development. Many types of cytokines and GFs contribute to proliferative phase, such as the transforming growth factor-β family [38,39], angiogenesis factor s (i.e., vascular epidermal GF), and interleukin family [40,41]. This phase can last for several days or even a few weeks. The last phase of the wound healing process is a remodelling phase which is also known as the maturation phase whereby the wound is fully closed and it takes place over weeks or months [42]. The surface of the injury is covered completely with fibroblasts as a new lining of the skin with a formation of a scar [43,44].

## 3. Classification of Wound Dressings

**Figure 2:**
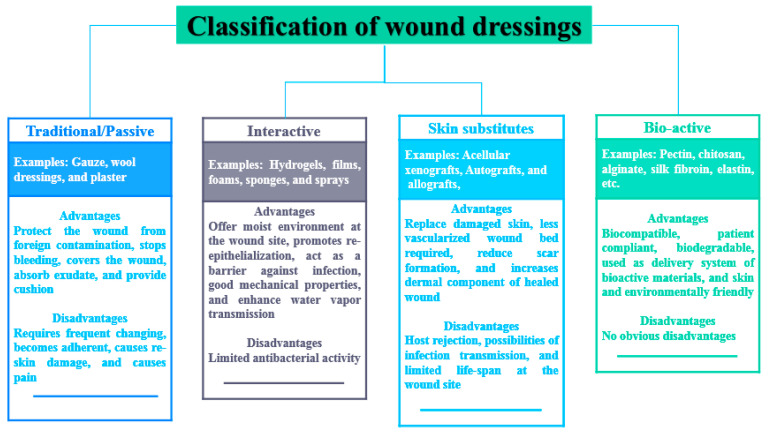
The classification of wound dressings.

Wounds, either acute or chronic, require appropriate management to combat various complications that may happen during the healing process. The dressings that are utilised for wound management are classified into four groups: traditional dressings, bioactive dressings, interactive dressings, and skin substitutes (Figure 2) [10,45]. Traditional wound dressings, also known as passive dressings, are responsible for the protection from foreign substances or contamination and injury. These dressings are also responsible for controlling bleeding, wound covering, absorption of wound exudate and cushioning the injury. Examples of traditional dressings include gauze and plaster [46]. The shortcoming of traditional wound dressings is that there is a need for frequent changing of the dressing during the healing process which can result in further skin damage [47].

Interactive wound dressings act as a gateway against microbial infections and offer moisture for the damaged skin, improving reepithelialisation and granulation. They also display good water vapor transmission rates [48]. These dressings are regularly synthesized from either synthetic or natural polymers such as PLGA, PLA, PGA, HA, gelatin, alginate, and chitosan [49]. Skin substitutes are wound dressings that are designed to substitute for the injured skin and they are composed of epidermal and dermal layers originated from keratinocytes and fibroblasts on a collagen matrix. There are several examples of skin substitute wound dressings such as autografts, acellular xenografts, and allografts [50]. The application of skin substitutes in the field of wound management is hampered by the limited time of survival on the wound environment, host rejection, and the possibility of transmission of infections [50].

Bioactive wound dressings are formulated for the delivery of bioactive molecules such as antibiotics. These dressings can also be encapsulated with antimicrobial agents, growth factors, and vitamins to enhance their therapeutic efficacy [34]. The examples of bioactive dressings include sponges, foams, wafers, hydrogels, films, membranes, nanofibers. These wound dressings are frequently designed from natural polymers or synthetic polymers such as chitosan, cellulose, silk fibroin, alginate, HA, pectin, elastin, etc. The properties that are demonstrated by bioactive dressings include good biocompatibility, biodegradability and their patient compliance [10]. Curcumin can be loaded in these wound dressing materials due to its several interesting therapeutic activities such as antibacterial and wound healing properties.

## 4. Biological Activity of Curcumin in Wound Healing

**Figure 3:**
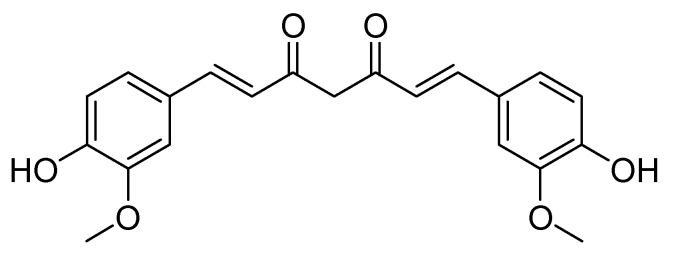
The structure of curcumin.

Curcumin is a natural herbal bioactive agent with anti-inflammatory, antibacterial and antioxidant properties [51]. It is a natural polyphenolic molecule (Figure 3) with potential wound-healing properties extracted from the *Curcuma longa* rhizome. The prospective wound-healing efficacy of curcumin is due to its anti-inflammatory, antibacterial, and antioxidant activity [52]. Oxidative stress is one of the factors that can result in slow wound healing process causing a wound to become chronic. Some antioxidant therapy is achieved by the encapsulation of antioxidant agents into wound dressing for targeting reactive oxygen species (ROS) whereby the eradication of ROS at the wound site could be considered as an important strategy to enhance the healing process of chronic wounds [53]. The *in vivo* experiments have demonstrated that curcumin enhance wound healing mechanism by decreasing ROS [54].

The development of ROS such as lipid peroxyl radicals, superoxide radicals, nitrogen dioxide radicals and hydroxyl radicals is related in the initiation of oxidative stress. A recent investigation utilizing animal prototypes revealed a significant defensive property of curcumin against the oxidative stress by scavenging the reactive free radicals involved in such a mechanism [55]. The phenolic hydroxyl groups contribute to its ROS scavenging ability and the diketone structure is considered to be responsible for its capability to attach to metals. The activation of the cytoprotective signaling constituents via the nuclear factor erythroid 2-related factor (Nrf2) pathway was identified as a related molecular mechanism responsible for the antioxidant efficacy of curcumin [55].

Curcumin also interacts with numerous molecular targets involved in inflammation. It reduces the transcription factor protein-1 (AP1) and nuclear factor kB (NFkB) expression that is responsible for regulating the expression of proinflammatory gene products and decreasing the expression of several inflammatory cytokines, such as interleukin (IL)-8, IL-6, IL-1, TNFα, migration inhibitory protein and monocyte chemoattractant protein and different chemokines [56,57]. Curcumin speeds up the healing process by acting on the inflammatory, proliferative and maturation phases of wound healing [55].

## 5. Curcumin-Loaded Wound Dressings

### 5.1. Hydrogels

**Figure 4:**
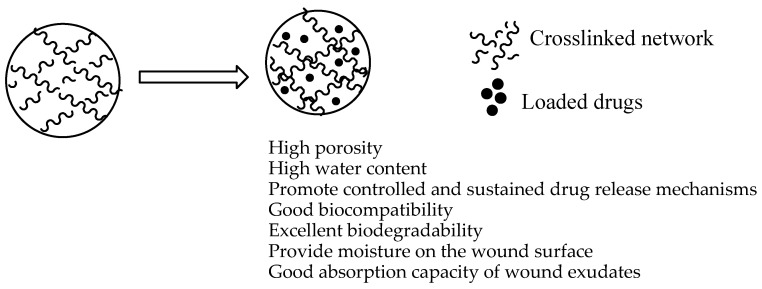
Hydrogel loaded with curcumin.

Hydrogels are 3-dimensional polymeric cross-linked wound dressings with hydrophilic properties and they possess the ability to absorb a huge volume of water and other biological fluids (Figure 4) [58]. These dressings have been utilized for wound healing because of their high porosity, high water content, and capacity to encapsulate and release therapeutic agents, good biocompatibility and biodegradability [59]. Although hydrogels display these interesting features, they suffer from several limitations such as their requirement for a secondary dressing, they can cause skin maceration, they can cause dehydration if not covered, exhibit poor mechanical properties in swollen state, etc. Their poor mechanical properties are usually overcome by preparing them from a combination of biopolymers and synthetic polymers, leading to hydrogels with excellent mechanical features [60]. Several researchers have reported polymer-based hydrogels loaded with curcumin.

Shefa et al. formulated oxidized cellulose nanofiber-PVA hydrogels incorporated with curcumin by a freeze-thaw method to promote the wound healing process [61]. The hydrogels were characterized by an interconnected micro- and macroporous network. The porosity of the hydrogels increased with an increase in the concentration of TEMPO-oxidized cellulose nanofiber (TOCN) used. The *in vitro* degradation studies showed that the hydrogels loaded with curcumin degraded by over 80% within 2 weeks when immersed in phosphate buffer saline solution (PBS) thereby releasing curcumin into the media. The biodegradation capability of the hydrogels promoted a sufficient amount of curcumin to be delivered into the skin resulting in accelerated wound healing. The cell viability analysis of the hydrogels on L929 cells using MTT assay demonstrated the non-toxic nature of the hydrogels. The *in vivo* wound closure experiments using full-thickness excision wounds in a rat model exhibited approximately 29.9% wound closure for the curcumin loaded polymeric hydrogels when compared to 8.3% for the control after 1 week. After 2 weeks, the hydrogels loaded with curcumin demonstrated a significant wound closure of 81.3% [61].

Pham et al. prepared thermal-responsive hydrogels from chitosan-Pluronic P123 loaded with curcumin and gelatin for wound healing [62]. The rheological analysis of the curcumin-loaded hydrogels and dual-loaded hydrogels demonstrated thermally-induced sol-gel transition feature. The swelling profile of the hydrogels showed a gradual increase in the swelling rate from 48 h to 400 h, reaching the maximum swelling of 268.51% at 432 h. The *in vitro* drug release studies showed an initial burst release from the hybrid hydrogels in the first two hours followed by a sustained drug release. The *in vivo* wound healing of the hydrogels encapsulated with gelatin possessed fast healing mechanism when compared to other groups. Furthermore, on day 18 the wound healing was approximately 99.76%. The hydrogel provided a moist environment which acts as the extracellular matrix suitable for cell migration into the hydrogel network thereby increasing epithelial cell migration and wound healing with reduced formation of scars [62].

Huang and co-workers formulated cellulose-halloysite nanotube hydrogel wound dressings for the delivery of curcumin. The addition of halloysite nanotube increased the viscosity of the hydrogels. The compressive strength of the hydrogels with 66.7% halloysite nanotube was 128 kPa, while that of the plain cellulose hydrogels was only 29.8 kPa showing that the mechanical properties of the hydrogels are improved by the addition of halloysite nanotube. The incorporation of halloysite nanotube in the hydrogel improved the drug-loading capacity and sustained-release effect. The SEM images displayed porous structure of the hydrogels with a pore size in the range of 200–400 µm. The XRD spectrums confirmed the amorphous nature of the biopolymeric hydrogels. The *in vitro* cytotoxicity studies of the hydrogels loaded with curcumin showed a high cell viability of MCF-7 and MC3T3-E1 cells after 2 days of incubation, confirming their good biocompatibility [63].

Anjum et al. designed novel nanosilver nanohydrogels that are based on polymethacrylic acid blended with a PEO/PVA/carboxymethyl cellulose matrix and loaded with curcumin and *Aloe vera* for wound dressing. The *in vitro* antibacterial analysis of the co-loaded nanohydrogels on *Escherichia coli (E. coli)* and *Staphylococcus aureus* (*S. aureus*) demonstrated an important bacterial reduction of over 80%, but it was lower than the single loaded nanohydrogels (revealing complete bacterial reduction of 100%) revealing the incompatibility of the drug combination. The *in vivo* wound healing studies using mice demonstrated that the wounds dressed with reference fabric possess only 60% wound healing after 16 days while dual drug-loaded nanosilver blended polymeric nanohydrogels showed 75% wound reduction [64]. Cirillo et al. prepared carbon nanotubes hybrid hydrogels from gelatin and PEG for electrically tunable release of curcumin [65]. The SEM images of hybrid hydrogels displayed the carbon nanotubes which were uniformly and randomly dispersed within the hydrogel matrix. The cytotoxicity analysis demonstrated that all the formulated curcumin loaded hybrid hydrogels possessed cell viability values of more than 94% on human fetal MRC-5 at all the evaluated concentrations that ranged between 0.1 and 1.0 mg/mL, revealing their excellent biocompatibility. The drug release profile displayed that a suitable amount of nanotubes in the hydrogels permitted the release for various therapeutic needs, including low and prolonged dosages at voltage condition of 0 V, fast release at 12 and 48 V, and intermediate behaviour at 24 and 36 V. All the above results revealed the interesting application of hybrid hydrogels for various therapeutic desires in wound treatment [65]. The distinct interactions between the drug and the hydrogel with the polarization of the hydrogel resulting from the electric stimulation allows modified a controlled release profile suitable for wound healing.

Gong and co-workers formulated PEG-poly (ԑ-caprolactone) (PCL)-PEG copolymer-based in situ gel-forming hydrogels loaded with curcumin and encapsulated micelles for cutaneous wound healing. The encapsulation efficiency and drug loading capacity of curcumin entrapped micelles was 98.40%, and 14.76%, respectively. These hydrogels were free-flowing sol at lower temperature but changed into non-flowing gel at physiological temperature of 37 °C. The *in vitro* drug release analysis showed a slow and sustained release of curcumin loaded micelles from the in situ gel-forming hydrogels. The wound healing analysis using rat linear excision wound model demonstrated a significant wound closure of approximately 94.47% for the hydrogels loaded with curcumin encapsulated micelles on day 14 when compared to the curcumin encapsulated micelles that displayed wound closure of about 82.93% ± 7.03%. The wound dressing was effective in the cutaneous wound healing process. The antioxidant activity of curcumin contributed to the decreased oxidative stress of the wounds via scavenging superoxide radical [66]. Alibolandi and co-workers prepared dextran hydrogels encapsulated with curcumin nanomicelles for full thickness wound treatment [67]. The swelling analysis showed a minor increase in the swelling index over a period of 8 days followed by a decreased swelling over 20 days, showing the ability of dextran hydrogels to absorb water over a period of 8 days. After 8 days, the dextran hydrogels degraded with the hydrolysis of the cross-linkages. The *in vivo* wound healing experiments on full thickness skin wounds of BALB/C mice showed that at day 21 after treatment, the wounds treated with curcumin nanomicelles loaded hydrogels were importantly reduced with almost complete skin regeneration. The sustained release of curcumin from the dressing resulted in decreased inflammatory responses, induced fibroblast proliferation and promoted collagen synthesis and angiogenesis in the healing process [67].

Li et al. designed in situ injectable hybrid hydrogels loaded with nano-curcumin from the oxidized alginate and N,O-carboxymethyl chitosan wound healing. The synthesized free nano-curcumin demonstrated very significant total antioxidant efficacy of approximately 99.22%. The *in vivo* wound repair evaluation showed that the application of nano-curcumin loaded hydrogel quickened the wound closure after 3 days of treatment when compared to the saline solution (reference) treatment. After 14 days of treatment, the wound area in the hybrid hydrogels/nano-micelles was reduced significantly when compared to the free drug, curcumin. The release of the nano-curcumin was slow and sustained from the hydrogel thereby stimulating collagen synthesis, fibroblast proliferation and capillary formation. Furthermore, the combination of the nanocurcumin with the hydrogel absorbed the growth factors, cytokines and enhanced the bioavailability of curcumin at the wound site [68]. Gupta et al. synthesized and evaluated bacterial cellulose hydrogels entrapped with curcumin and they were loaded in the cyclodextrins for wound treatment. The SEM images of the hydrogels displayed dense interwoven fibre network with pore size of 27 nm. The water content of the cellulose hydrogels was high. DPPH assay was used to determine the antioxidant activity of the hydrogels and the bacterial cellulose hydrogels loaded with curcumin displayed decreased oxidative stress at the wound site [69].

Du et al. formulated poloxamer-based hydrogels encapsulated with curcumin-phospholipid complex for wound healing. The hydrogels loaded with curcumin-phospholipid complex exhibited higher erosion rates when compared to the curcumin-loaded hydrogels because of the amorphous nature of the curcumin-phospholipid complex, which can result in an increase in curcumin dissolution. The *in vivo* wound healing experiments of curcumin-phospholipid complex loaded poloxamer-based hydrogels exhibited a higher healing effect in comparison with the reference on the rat skin wound model [70]. Kumar et al. designed curcumin nanoparticles loaded hydrogels that are based on oxidized alginate-N,O-carboxymethyl chitosan for diabetic skin wound healing. The *in vivo* wound closure experiments revealed that the diabetic wounded rats treated with curcumin nanoparticles-loaded hydrogel displayed a higher rate of wound closure of 93.3% when compared to the free curcumin that displayed a 58.3% wound closure. The enhanced healing of the diabetic skin wound was via increased rate of wound closure, high formation of granulation tissue, deposition of collagen, high production of vascular endothelial growth factor (VEGF) and expression of Aquaporin 3 (AQP3) [71]. Ravindra and co-workers prepared poly (acrylamide-co-acrylamido-propano sulphonic acid) hydrogels encapsulated with curcumin loaded silver nanoparticles. The swelling ratio of the drug-loaded hydrogels was high. The *in vitro* antibacterial studies of the hydrogels encapsulated with curcumin loaded silver nanoparticles displayed superior bacterial inhibition against *E. coli* on the nutrient agar medium when compared to the plain hydrogels, revealing their efficacy against microbial infected wounds. The loaded curcumin silver nanoparticles in the hydrogels influenced the antibacterial activity of the hydrogel [72].

Zhao et al. formulated thermosensitive β-glycerophosphate/chitosan hydrogels encapsulated with β-cyclodextrin-curcumin for cutaneous wound infection treatment. The *in vitro* drug release analysis demonstrated that curcumin was released at a slow sustained pattern of 39.95% within 2 weeks. *In vitro* degradation behaviour studies showed that 53.26% of the hydrogel degraded within 2 weeks. The wound healing studies showed that the β-glycerophosphate/chitosan hydrogels encapsulated with β-cyclodextrin-curcumin revealed a faster healing rate of 94.67% ± 2.29% on day 14 when compared to the plain hydrogels that displayed a healing rate of 85.84% ± 8.83% on day 14 and the gauze healing rate was 18.17% ± 9.83%. The drug-loaded hydrogel suppressed the NF-κB signaling pathway. The oxidative stress of the hydrogel was reduced due to the ROS scavenging capacity of curcumin. The combination of chitosan, an immunomodulatory compound with curcumin reduced the inflammatory responses to the microbial infection [73]. Li et al. designed in situ gel-forming hydrogels co-encapsulated with curcumin and epidermal growth factor (EGF) for skin regeneration. The *in vivo* wound healing experiments demonstrated that the dual-loaded hydrogels significantly improved wound closure on excisional full-thickness wound model via increasing collagen deposition, angiogenesis, and granulation tissue development when compared to the normal saline and single bioactive agent loaded hydrogel. In addition to accelerated closure, the hydrogel also promoted the enhanced biosynthesis of TGF-β1 and new vessel formation in the wounds [74].

Rezvan and co-workers formulated curcumin-loaded Pluronic F127 nanomicelles encapsulated PVA-borax dual delivery polymeric hydrogels for wound dressing. The *in vitro* drug release studies using UV-vis spectroscopy analysis demonstrated that these hydrogels are appropriate scaffolds for curcumin delivery for wound healing applications [75]. Juan and co-workers synthesized hydrogels loaded with gelatin microsphere containing curcumin nanoparticles for diabetic wound healing. The drug release profile of the hydrogels induced curcumin release at the wound bed thereby stimulating the healing of the streptozotocin-induced diabetic mice skin wounds [76]. Zhang et al. synthesized in situ forming PVP-based hydrogels encapsulated with curcumin solid dispersion for vaginal wound healing and vaginal bacterial infection treatment. These scaffolds possessed the ability to form a gel in the vagina environment. The *in vitro* antibacterial studies of curcumin-loaded hydrogels demonstrated good antibacterial efficacy against bacterial strains that can cause microbial infection in the vagina injury (such as *E. coli* and *S. aureus*). The *in vivo* wound healing studies demonstrated that these hydrogels enhanced rat vaginal wound contraction by promoting inflammation and repairing vaginal epidermal tissues [77].

### 5.2. Films/Membranes

Film wound dressings are usually made up of adherent and transparent polyurethane that allows the transmission of water vapor water, carbon dioxide, and oxygen between the wound and the external environment [78,79]. These dressings also improve the wound healing process and protect the injury from microbial infections [80]. The wound healing process can be monitored without the removal of the film because of its transparency. Hence, they are ideal dressings for superficial wound, epithelizing wound, and shallow wound [81]. The other interesting properties of the currently employed films include their high elasticity and flexibility resulting in their ability to be manipulated to any form with no need for additional tapping [82,83]. However, film dressings are not suitable for high exudate wounds because of their inability to absorb high amounts of biological fluids. Li et al. formulated methoxy PEG-g-chitosan composite films encapsulated with a designed curcumin nanoformulation for wound healing applications. The TEM images of the designed curcumin nanoformulation demonstrated spherical shapes with a mean diameter of about 40 nm. The SEM images showed that the cross-section films was approximately 30 µm with no clear separation of curcumin showing that they appropriate for the wound healing. The *in vivo* wound closure of the plain polymeric films was approximately 62% on day 3 of post wounding, whereby the wound reduction from the curcumin loaded films was over 80% on the same day [84].

Dhurai et al. formulated electrospun PLA/chitosan nanofilms loaded with curcumin for biomedical application. The energy dispersive X-ray (EDX) and FTIR analysis confirmed the successful formulation of drug loaded nanofilms. The *in vitro* cytotoxicity studies showed that the non-reactivity of nanofilms to fibroblast cells after 24 h of incubation revealed their good biocompatibility. The *in vivo* wound healing tests of the curcumin entrapped nanofilms on incision and excision wounds made on the rat models revealed a significant wound closure when compared to the untreated wounds [85]. Khamrai et al. formulated gelatin/ bacterial cellulose-based hydrogel films entrapped with curcumin [86]. The self-healing capability of the wound dressings was significant. The mechanical properties of the hydrogel films were 2.5% elongation with a modulus of 6 MPa at the breaking point, whereas the composite hydrogel shows an elongation of 4.8% and a modulus value of 4.6 MPa. The *in vitro* antibacterial analysis showed that curcumin-loaded hydrogel films effectively inhibited the bacterial growth with a zone of inhibition value of 15 ± 0.5 mm against *E.coli*, and 19 ± 1.0 mm against *S. aureus*. The *in vitro* wound healing analysis showed that the hydrogels films loaded with a higher curcumin loading accelerated the cell migration and proliferation. The curcumin-loaded hydrogel films healed the wound completely when compared to the references that healed up to 59% [86].

Tong et al. designed antimicrobial cellulose nanocrystals film wound dressings loaded with curcumin. The TEM results displayed needle-like cellulose nanocrystals with a mean length of approximately 159 nm. The cellulose nanocrystals films encapsulated with curcumin was flexible and soft. The antimicrobial analysis of the films demonstrated significant inhibitory efficacy against *E. coli*, *Yersinia sp*. *Proteus mirabilis*, and *Pseudomonas aeruginosa.* The *in vivo* wound analysis utilizing diabetic rat models showed a reduction of the wound area on day 7 with the topical treatment of films loaded with curcumin. Curcumin accelerated the wound healing by increasing the level of TGF-β1 which is useful for cell growth and proliferation. Curcumin also protects the skin cells from oxidative damages [87]. Wathoni and co-workers formulated 2-hydroxypropyl-ϒ-cyclodextrin sacran hydrogel films incorporated with curcumin for wound healing application. The *in vitro* drug kinetics release of curcumin from the films at physiological conditions (pH 7.4 and temperature of 37 °C) was a biphasic release with an initial release of 49.69% of curcumin in the first 24 h, followed by a rapid release of 69.40% until 120 h. The *in vivo* wound healing evaluation using hairless mice full-thickness excisional wounds showed that the curcumin loaded 2-hydroxypropyl-ϒ-cyclodextrin sacran hydrogel films enhanced wound repair capacity from day 3 to 14 days [88].

Manna et al. developed curcumin encapsulated carboxymethylated guar gum-g-gelatin film dressings for biomedical application. The mechanical property analysis of films showed tensile strength of 41.64 MPa, while the tensile strength of CMGG and native gelatin strength was only 26.07 and 3.35 MPa, respectively. The antibacterial analysis of the films demonstrated good growth inhibition against *E. coli*, *E. aerogenes*, *Lysinibacillus* and *S. aureus* with the average diameter of inhibition zone ranging between 12 ± 0.5 and 17 ± 1.0 mm. The above results demonstrated the interesting benefit of curcumin in wound healing [89]. Bajpai and co-workers formulated nanocellulose dispersed chitosan film wound dressing loaded with curcumin/Ag nanoparticles [90]. The wound healing analysis of drug dual-loaded polymeric films showed significant wound closure when compared to the single drug-loaded films, while combination of drugs revealed significant enhancement in the wound healing capacity. Bajpai and co-workers also formulated cellulose/chitosan microcrystals antimicrobial films loaded with curcumin for wound dressing. The *in vitro* drug release profile demonstrated that the amount of curcumin released decreased with increase in the cellulose concentration of the polymeric films. The *in vitro* antimicrobial studies showed that the curcumin-loaded films revealed inhibition zones of nearly 3 cm for *C. albicans*, and 3.5 cm for *C. parapsilosis* [91].

Varaprasad et al. synthesized curcumin impregnated with silver nanocomposite films for antibacterial applications. The *in vitro* antibacterial analysis of the dual-loaded films showed significant growth inhibition of *E. coli* strain when compared to Ag nanoparticles or curcumin single loaded films revealing the efficacy of combining curcumin and silver nanaoparticles. Curcumin suppresses the growth of bacteria and controls the release of silver nanoparticles from the films networks resulting in significant antibacterial activity [92]. Naseri-Nosar and co-workers formulated curcumin-encapsulated chitosan microparticles loaded into electrospun PLA-based films for wound dressing. The *in vitro* cytotoxicity of the polymeric films exhibited excellent cytocompatibility to L929 fibroblastic cells. The *in vivo* wound healing analysis of curcumin-encapsulated chitosan microparticles loaded into electrospun PLA-based films employing full-thickness excisional wounds of rats showed significant wound closure percentage when compared to plain films and sterile gauze. A synergistic effect of curcumin and chitosan resulted in accelerated healing attributed to a high number of transforming growth factor-β in fibroblasts which are useful for the synthesis of collagen bundles cross-linking and improved the wound contraction [93]. Vimala and co-workers synthesized chitosan-PVA silver nanocomposite films loaded with curcumin. The *in vitro* antimicrobial analysis of the chitosan-PVA silver nanoparticle films loaded with curcumin exhibited important effects against *E. coli*, *Staphylococcus*, *Pseudomonas*, *Micrococcus*, *Candida albicans*, and *P. aeruginosa*. Furthermore, these curcumin loaded films revealed enormous inhibition of *E. coli* growth when compared to the free curcumin and plain chitosan-PVA silver nanoparticles films. These results clearly revealed the efficacy of combining silver nanoparticles with curcumin in novel antibacterial films which are potentially useful in microbial infected wounds [94].

Liu et al. formulated chitosan films encapsulated with curcumin-loaded PLGA-chitosan microspheres for skin regeneration [95]. The atomic force microscopy (AFM) images of the films demonstrated 3D morphologies with different degrees of surface roughness. The *in vitro* drug release mechanism at physiological conditions revealed a first burst release of curcumin from the chitosan membranes followed by a sustained drug release. The mechanical analysis of curcumin-loaded films showed a significantly higher tensile strength, less Young’s modulus which may be caused by the intermolecular interaction between curcumin and chitosan. The polymeric films loaded with curcumin completely inhibited bacterial growth of *S. aureus* and *E. coli* after 12 h of treatment when compared to the plain films *in vitro*. *In vivo*, the wound healing profile of the curcumin encapsulated films using full-thickness size skin wound on a rat model was faster when compared to the reference gauze group after a week [95]. Ponnanikajamideen and co-workers formulated chitosan-PVP nanofilms loaded with curcumin for wound healing capacity. The curcumin-loaded films showed mechanical properties that were more appropriate for the management of wound healing. The number of pores on the curcumin-loaded films were high and they exhibited a high swelling capacity when compared to the plain chitosan-PVP nanofilms making them useful for topical wound healing application [96].

Reshmi et al. formulated nano-chitosan loaded poly ԑ-caprolactone membranes loaded with curcumin for antibacterial wound dressings [97]. These electrospun membranes displayed a significant increase in the fiber mean diameter up to 828 ± 94 nm. The XRD and FTIR spectra demonstrated amorphous nature and the expected functional groups of the polymeric membranes, respectively. The tensile strength of the membranes was in the range of 7.9 ± 0.1 N/mm^2^ to 3 ± 0.3 N/mm^2^, elongation percentage of the scaffolds from 65% to 39%, and the Young’s modulus values ranged from 0.12 N/mm^2^ to 0.07 N/mm^2^. The electrospun polymeric membrane demonstrated good porosity of 75% and mean pore diameter of 3397 ± 341 nm due to the development of uniform smaller diameter fibers. The *in vitro* drug release profile of PCL electrospun membrane at pH 7.4 demonstrated only 36 % release of curcumin over a period of 15 days. The antibacterial efficacy against *S. aureus* increased with increase in the addition of nanochitosan [97]. Baldino and co-workers synthesized biodegradable cellulose acetate-based membranes loaded with curcumin. *In vitro* antioxidant analysis revealed that the significant antioxidant activity of the loaded curcumin was retained, which was influenced by the films pore size. The control release of curcumin from the films enhanced its shelf-life *in vitro* [98].

Marulasiddeshwara and co-workers formulated chitosan-based membranes impregnated with curcumin/TiO_2_ complex for Methicillin-resistant Staphylococcus aureus (MRSA) infected wound skin regeneration [99]. The water uptake of the films were high with approximately 480.8%, whereas the curcumin-loaded chitosan membranes exhibited a lower water uptake capability of over 186% which is caused by the hydrophobic nature of curcumin. The chitosan-based membranes demonstrated a low bactericidal activity and the addition of curcumin increased the zone of inhibition to 4 mm against *E. coli* and *S. aureus*. The *in vivo* studies of the curcumin-loaded membranes demonstrated a gradual increase in wound closure. Furthermore, a methicillin-resistant *Staphylococcus aureus* (MRSA)-infected wound treated with chitosan membranes co-loaded with curcumin and TiO_2_ revealed enhanced wound contraction on day 14 due to the synergistic effect of combining both therapeutic agents [99]. Cardea et al. designed polyvinylidene fluoride-hexafluoropropylene (PVDF-HFP)-based membranes encapsulated with curcumin. The porosity analysis showed that the average pore size of drug-loaded polymeric membranes ranged between 6.0 ± 2.3 μm and 20.0 ± 4.2 μm. The mechanical experiments of PVDF-HFP-membrane loaded with curcumin showed a significant tensile strength of 1.58 MPa revealing that the encapsulation of curcumin did not significantly influence the mechanical properties of the formulated membranes. Furthermore, the antioxidant analysis showed that the antioxidant activities of curcumin-loaded PVDF-HFP membranes ranged between 76%–90% [100].

### 5.3. Sponges/Bandages

Sponges are soft and flexible wound dressing materials that possess interconnected porous structures [101]. Their porous structure provides various advantages including high hemostatic capacity, high swelling capacity, and high water absorption ability with the capacity to offer a moist environment for the wound and protect the wound from microbial infections [102]. The sponges that have pore sizes ranging between 10 and 100 µm with interconnected structures possessed the ability to promote cell adhesion and proliferation [103]. Polymeric sponges formulated from materials such as PVA, cellulose, sodium alginate, chitosan, and graphene oxide, demonstrate good antibacterial properties. Sponges formulated from biopolymers display good antimicrobial properties, intrinsic hemostatic capability, good biocompatibility, and moderate water vapor transmission rate [104]. These interesting properties make biopolymer-based sponges promote their selectivity against certain strains of bacterial, dehydration and gas permeation [105].

Nguyen and co-workers formulated gelatin/chitosan composite sponges encapsulated with curcumin for wound healing application. The expected chemical functional groups were demonstrated by FTIR. The SEM images of sponges exhibited a porous structure with rough surfaces. The pore sizes ranged between 29.9–43.06 μm. Sponges prepared with a low concentration of gelatin displayed regular pore sizes suitable for gaseous exchange and the transmission of nutrients to the wound. The water uptake analysis of the plain sponges demonstrated water uptake efficiency of 2352%, 351%, and 227%, for ratios of gelatin/chitosan of 1:3, 1:1, and 3:1, respectively. The water absorption efficiency of curcumin-encapsulated sponges was not significantly different from the plain polymeric sponges [106]. The *in vitro* release kinetics of curcumin increased with an increase in the amount of gelatin in the formulated sponges. The *in vitro* antibacterial analysis of curcumin-loaded polymeric sponges revealed a higher inhibition zone that ranged between 21–17.5 mm against *Pseudomonas aeruginosa* bacteria when compared to the plain gelatin/chitosan composite sponges. The cytotoxicity evaluation of all sponges employing MTT assay method demonstrated significant high cell viability of 90% on L929 firoblast cells within 24 h of incubation. The *in vivo* wound healing using excision wound model in albino rabbits demonstrated that the wound treated with curcumin-loaded gelatin/chitosan composite sponges showed a healing of 99.49% after 21 days when compared to 97.63% wound closure for the plain sponges [106].

Momin and co-workers formulated alginate-chitosan hydrogel composite sponges co-encapsulated with curcumin and honey via in situ polymerization method for potential application in wound management. The swelling analysis of the biopolymer sponges demonstrated a high swelling capacity of 111.05%. The tensile strength of the sponge was 4323 gm/mm^2^. The *in vitro* drug release profile demonstrated slow and sustained release of curcumin from the sponges, showing that only 75.03 ± 3.59% of curcumin was released within 20 days. The wound healing assessment of alginate-chitosan hydrogel sponges co-encapsulated with curcumin and honey demonstrated significantly accelerated wound closure of 94.14% in one week revealing them as potential wound dressings for pressure ulcers or diabetic foot [107]. Zhao et al. prepared curcumin-loaded β-cyclodextrin complex and then encapsulated it in chitosan-alginate sponges. The SEM analysis demonstrated that the sponges possessed porous morphology. The water uptake evaluation revealed the superior water uptake capability of the polymeric sponges which could be due to their porous structure. The *in vitro* drug release profile of curcumin was sustained within 2 weeks with over 60.61% of curcumin released from the sponges. The *in vivo* wound healing studies on rat model showed the fastest healing of 94.39% for wounds treated with curcumin-loaded sponges over a period of 14 days when compared to 64.55% wound healing for gauze [108].

Mohanty et al. prepared oleic acid based chitosan-alginate bandages loaded with curcumin for applications in wound healing. The SEM images of polymeric bandages were fibrillar and porous morphology. The water uptake and degradation analysis at pH 7.4 showed superior water uptake and excellent degradation features for the formulated chitosan-alginate bandages. The *in vitro* drug release profile showed that curcumin was released from oleic acid-based polymeric bandages in a sustained manner, a feature suitable for perfect drug dosing thereby improving the bioavailability of curcumin at the wound site. The *in vivo* wound healing studies using wounds on Sprague Dawley male mice demonstrated that wounds treated with oleic acid based chitosan-alginate bandages loaded with curcumin were healed by 94% when compared to 70% wound closure for cotton gauze (control) after 10 days of treatment [109].

Mohantya and Pradhan formulated chitosan-alginate bandages co-encapsulated with curcumin and human epidermal growth factor (EGF)/mesenchymal stem cells (MSCs) for application in diabetic wound healing [110]. The atomic force microscopy (AFM) and SEM results demonstrated that the formulated sponges possessed sponge-like with microporous morphology beneficial for the transportation of oxygen and nutrients to support mesenchymal stem cell attachment and proliferation. The water contact angle was 64.82° and 76.89° for curcumin-EGF loaded sponges and EGF-loaded sponges, respectively, suggesting the good hydrophilicity of the chitosan-alginate bandages because their contact angles were less than 90°. The *in vitro* drug release profile was an initial burst release of 52% in 2 days followed by a sustained drug release of 84% in 15 days. The wound closure of drug-loaded polymeric sponges on diabetic rat model was prominent with good re-epithelialization on 12th day when compared to the untreated wounds [110].

### 5.4. Nanofibers

Nanofiber wound dressings have the capability to stimulate hemostasis of damaged tissues, cell respiration, support dermal drug delivery, enhance fluid absorption, and promote high-gas permeation, thereby inhibiting microbial infections [111]. The properties of nanofibers also include their capability to deliver bioactive agents, possess high surface area-to-volume ratio, exhibit improved mechanical properties and high porosity [112]. Electrospinning method is one of the best and efficient techniques that is currently employed to formulate polymeric nanofibers. There are several interesting advantages of electrospinning technique including controlling of the nanofiber composition to accomplish the desired feature, reducing the side effects of the systemic treatments etc. [113]. The loading of biological agents into nanofibers can be achieved due to their high surface-to-volume ratio. However, the electrospinning of some polymers suffers some obstacles such as low mechanical strength, low solubility and polycationic nature in solution [114].

Merrell et al. prepared PCL-based nanofibers loaded with curcumin for diabetic wound treatment [115]. The development of beads along the nanofibers was influenced by the concentration of PCL used for the preparation of the nanofibers. Electrospinning method using 15% (*w/v*) PCL resulted in nanofibers with an average diameter ranging between 300 and 400 nm. The *in vitro* drug release kinetics at physiological conditions of the curcumin from the nanofibers was sustained for 3 days and could be formulated to transport an amount much lower than the known cytotoxic concentration while remaining therapeutically active. The *in vitro* cytotoxicity studies displayed that the cell viability of the human foreskin Fibroblast (HFF-1) cells was more than 70% confirming the non-cytotoxic effect of curcumin-loaded PCL nanofibers. The inflammatory studies using the prepared nanofibers incorporated with curcumin decreased inflammatory induction from the rat monocyte macrophages. The *in vivo* wound healing experiment of the curcumin loaded PCL nanofibers loaded with curcumin exhibited an accelerated 80% wound closure in the streptozotocin induced diabetic rat model, while plain PCL displayed only a 60% wound closure [115]. Ramalingam and co-workers formulated electrospun poly(2-hydroxyethyl methacrylate) (p(HEMA)) nanofibers loaded with curcumin. The *in vitro* drug release profile of curcumin-loaded nanofibers demonstrated controlled and sustained curcumin release which was effective against the wound microbial infections. The *in vitro* antibacterial analysis of curcumin-loaded p (HEMA) nanofibers exhibited higher growth inhibition against extended spectrum β-lactamase (ESBL) and MRSA [116].

Nguyen et al. prepared curcumin-loaded PLA nanofibers for wound management. The encapsulation of curcumin into the nanofibers resulted in a significant increase in the tensile stress up to 3.5 MPa which is suitable for wound dressing. The *in vivo* wound healing studies on dorsal wounds of rats revealed 87% and 99% wound closure on 7th day and 15th day, respectively [117]. Ravikumar and co-workers formulated electrospun cellulose acetate phthalate polymer nanofibers loaded with curcumin. The swelling analysis demonstrated that the curcumin-loaded nanofibers and plain nanofibers revealed 400% swelling capacity between 1 and 12 h. The *in vitro* diffusion analysis showed a slow and sustained release of curcumin which is important for wound healing [118].

Ranjbar-Mohammadi and co-workers formulated PCL/gum tragacanth electrospun nanofibers loaded with curcumin. The PCL/gum tragacanth nanofibers loaded curcumin was 85.14% and 99.9% effective against ESBL and MRSA, respectively revealing that are useful for the treatment of bacterial infected wounds. The *in vivo* wound healing experiments using wounded diabetic Sprague Dawley rats showed that the wound areas covered with PCL/gum tragacanth nanofibers loaded with curcumin were completely closed on day 15 when compared to the control in which the wound area was reduced by 20.96% [119]. Furthermore, Ranjbar-Mohammadi and Bahrami reported curcumin loaded nanofibers with excellent biological properties. The nanofibers were bead free and the loading of curcumin provided a hydrophilic surface useful for cell attachment and proliferation. It also improved the mechanical properties of the nanofibers with the tensile strength which was over 2–3 folds increase. The stability of the nanofibers was also enhanced by the presence of curcumin. The nanofibers promoted significant cell growth and proliferation with retained morphology for a period of 15 days. The *in vitro* curcumin release studies from the nanofiber was sustained [120]. Ghaee et al. designed PCL-based nanofibers encapsulation with curcumin and incorporated in gelatin/chitosan for skin regeneration. The porosity of the nanofibers ranged between 90.43% and 71.48% with a pore size of 101–256 μm suitable for skin regeneration. The nanofibers were biocompatible on L929 cells and promoted good cell attachment [121].

Moradkhannejhad et al. formulated curcumin-loaded PLA/PEG nanofibers with a porous nanostructure morphology useful for gaseous exchange. The average diameter of the fibers was in the range of 430–750 nm which increased as the concentration of PEG1500 increased from 0 to 20 wt%. The nanofibers displayed controlled release of curcumin [122]. Mutlu et al. formulated electrospun poly (3-hydroxybutyric acid-co-3-hydroxyvaleric acid) (PHBV)-based nanofibers loaded with curcumin [123]. The mean fiber diameters of the nanofibers ranged between 207 and 519 nm, depending on the concentration of curcumin. The tensile strength and elastic modules was 5.80 MPa and 6.10 MPa, respectively. The swelling ratio of the nanofibers increased from 50% to 320% after the loading of curcumin. It was biocompatible with L929 mouse fibroblasts and promoted the cell attachment and proliferation *in vitro* [123].

Bui and co-workers developed PCL-PEG nanofibers incorporated with curcumin for enhanced wound healing. The prepared nanofibers possessed porous surface important for cell proliferation. The curcumin-loaded nanofibers displayed superior growth inhibition against *S. aureus* when compared to the plain nanofibers. The nanofibers loaded with curcumin accelerated the rate of wound closure by 99% on day 10 when compared to the plain PCL-PEG nanofiber with a 59% wound closure [124]. Mohammadi and co-workers formulated PCL-PEG nanofibers loaded with chrysin-curcumin for wound healing. The *in vivo* studies on wounded male rats demonstrated that the wound-healing process was dose-dependent that significantly affected the inflammation phase when compared to other phases of the wound healing. An elevated IL-6 gene expression was observed after 10 days *in vivo* and it plays an important role in inflammation. A reduced MMP-2 expression and a downregulation of iNOS was observed [125]. Perumal et al. prepared electrospun PLA-hyperbranched polyglycerol-based nanofibers loaded with curcumin [126]. The fiber diameter was 601 nm and the mean diameter of the nanofibers increased due to the loading of curcumin into the nanofibers. The nanofibers were hydrophilic enhancing controlled drug release, good cell proliferation and adhesion when compared to the nanofibrous PLA alone. The swelling ratio of the nanofibers reached 108% within 24 h. The *in vitro* drug release profile at physiological condition was an initial burst release followed by a controlled release pattern. The *in vitro* cell viability analysis of curcumin loaded nanofibers using Swiss 3T3 fibroblast cells exhibited significantly higher cell viability of 109% when compared with 96% of control and 100% for the plain nanofibers. The curcumin-encapsulated PLA-hyperbranched polyglycerol-based nanofibers displayed a 100% wound closure after 36 h of treatment when compared to curcumin-loaded PLA nanofibers [126].

Rramaswamy and co-workers developed PCL-PEG electrospun transdermal nanofibers loaded with tetrahydro curcumin. These nanofibers demonstrated a high entrapment efficiency of 95% of curcumin incorporation into the nanofibers which was due to the high surface area. The swelling capability was 205% for curcumin-loaded nanofibers and 215% for the plain nanofibers, showing decreased swelling ability after the addition of curcumin. The *in vitro* drug release profile of curcumin from the nanofibers was a sustained release [127]. Shababdoust and co-workers formulated amphiphilic-block segmented polyurethane-based nanofiber for controlled release of curcumin [128]. The porosity ranged between 80.1% ± 0.5% and 91.6% ± 0.4% with the average diameter ranging between 651 ± 209 and 663 ± 300 nm. The porosity and diameter of the nanofibers were influenced by the amount of the loaded curcumin. The *in vitro* antibacterial experiments demonstrated a high antibacterial efficacy of the nanofibers against *S. aureus* and *E. coli*. The curcumin-loaded nanofibers exhibited cell viability ranging between 89% and 92% on the L929 fibroblast cells, revealing their biocompatibility for the wound site. The *in vitro* drug release profile of curcumin from the polymeric nanofibers was influenced by the temperature, pH, and pressure [128].

Other nanofibrous materials are wound dressings that demonstrate almost the same properties and advantages as nanofibers. These dressings are also frequently formulated by electrospinning method. The examples of nanofibrous materials include nanofibrous mats [129,130,131], nanofibrous films, nanofibrous patches, nanofibrous membranes, etc. [132,133,134]. Fu et al. prepared PCL-PEG nanofibrous mats loaded with curcumin for dermal wound healing application [135]. Their diameter ranged between several hundred nanometers and few microns. When incubated with rat fibroblast cells, the nanofibrous membranes displayed high cell viability revealing their low toxicity. The *in vitro* drug release profile of curcumin was an initial burst release followed by a sustained drug release profile. The *in vivo* dermal wound healing experiments indicated a significant wound closure of 93.3% on day 21 for the curcumin-loaded nanofibrous mats when compared to 76.9% and 80.4% wound closure for the control (untreated wounds) and plain nanofibrous mats, respectively [135].

Lian et al. formulated silk fibroin/PLA-PCL nanofibrous scaffolds loaded with curcumin [136]. The mean nanofiber diameter of 461 ± 215 nm gradually decreased to 293 ± 110 nm after the addition of curcumin. The average tensile strength was 5.27 ± 0.34 MPa and elongation at break of 117.4 4 ± 1.35%. The *in vitro* drug release profile showed a burst release of curcumin from the scaffolds during the first 12 h followed by a sustained release for 72 h. The *in vitro* antioxidant studies of curcumin-loaded nanofibrous scaffolds employing 2,2-diphenyl-1-picrylhydrazyl (DPPH)-free radical scavenging assay showed a significant scavenging efficacy which increased gradually with an increase in the amount of curcumin that ranged between 2.0% and 6.0% (*w/w*), confirming the excellent antioxidant activity of the scaffolds. The curcumin-encapsulated nanofibrous scaffolds possessed high growth inhibition of 99.7% ± 0.85% against *S. aureus* when compared to 15.8% of plain nanofibrous [136].

Tsekova and co-workers formulated cellulose acetate/PVP electrospun fibrous materials loaded with curcumin for bacterially-infected wounds. The viscosity analysis of the cellulose acetate/PVP solutions loaded with curcumin in water/acetone showed significant increased viscosity of 142 cP resulting from the hydrogen bonds between the curcumin and polymers. The water contact angle of the curcumin-loaded nanofibrous scaffolds was 121.8° ± 3.4°. The *in vitro* microbial analysis of nanofibrous materials loaded with curcumin displayed good antibacterial activity on *S. aureus*, suggesting that these scaffolds are effective for the management of bacterial infection wounds [137]. Celebioglu and Uyar prepared hydroxypropyl-γ-cyclodextrin/hydroxypropyl-β-cyclodextrin-based nanofibrous scaffolds encapsulated with curcumin. The nanofibrous scaffolds demonstrated a bead-free morphology with uniform fibrous structure. The average diameter of the nanofibrous scaffolds was 165 ± 65 nm. The encapsulation efficiency (%) of curcumin was 98.8% ± 1.6% and 99.3% ± 1.0% in the hydroxypropyl-γ-cyclodextrin and hydroxypropyl-β-cyclodextrin nanofibers, respectively. The antioxidant analysis of curcumin-loaded nanofibrous material using DPPH scavenging assay revealed significant high antioxidant efficacy of 100% for the curcumin-loaded hydroxypropyl-gamma-cyclodextrin webs when compared to 34.7% for the hydroxypropyl-β-cyclodextrin. The curcumin-loaded hydroxypropyl-γ-cyclodextrin nanofibrous webs are potential wound dressing [138].

Saeed and co-workers prepared PCL/PVA electrospun three-layer nanofibrous mats loaded with curcumin for active wound healing. The water contact angle and the water vapor transmission test demonstrated a higher water vapor transmission rate (WVTR) for the three-layer nanofibrous mats due to the hydrophilicity nature of the PVA layers when compared to the monolayer mat (control). The *in vitro* antibacterial evaluation of the multi-layer electrospun mats displayed a higher percentage of bacteria reduction against *E. coli* and *S. aureus* after 2 days of incubation. The properties of the three-layer nanofibrous mats loaded with curcumin are potential materials for wound healing application [139]. Esmaeili et al. formulated PU/cellulose nanofibrous mats co-encapsulated with silver nanocomposites/graphene oxide and curcumin for wound dressing. The *in vitro* antimicrobial analysis of the drug co-loaded nanofibrous mats demonstrated a high synergistic antibacterial activity against *S. aureus* and *Pseudomonas* bacteria when compared to the drug-loaded mats. *The in vivo* wound closure experiments of dual drug-loaded polymeric nanofibrous mats demonstrated a significantly accelerated wound healing rate resulting in a 100% wound healing when compared to 78% for the control (plain nanofibrous mats), 90% for the graphene oxide-loaded mats and 93% for the Ag loaded mats [140].

Pankongadisak et al. formulated PLA-based electrospun nanofibrous mats loaded with curcumin as wound dressing scaffolds [141]. The TEM analysis showed that the loading of curcumin in the nanofibrous mats decreased the average diameter of the plain electrospun mats from 386 ± 121 nm to diameter ranging between 333 ± 124 and 380 ± 113 nm. The mechanical property analysis demonstrated that curcumin-loaded nanofibrous mats possessed tensile strength of 2–3 MPa, elongation at break of 40–49%, and Young’s modulus of 57–111 MPa. The drug release profile *in vitro* at physiological condition showed that curcumin was initially released from the nanofibrous mats followed by a sustained drug release after an hour. The antioxidant evaluation using DPPH assay demonstrated the antioxidant efficacy that ranged between 42.50% and 52.96% for electrospun polymeric fiber mats loaded with curcumin suggesting their good antioxidant effect for wound dressing applications [141]. Mahmud and co-workers designed electrospun fiber mats loaded with curcumin for antibacterial wound dressings [142]. The *in vitro* drug release profile of curcumin was temperature-dependent and controlled from the nanofibrous mats. The swelling analysis of the mats demonstrated a swelling capacity of 332%. The *in vitro* antibacterial tests exhibited 100% reduction against *S. aureus* and *E. coli* bacteria after 6 h of incubation. Cellulose acetate electrospun fiber mats loaded with curcumin formulated by Suwantong and co-workers demonstrated antioxidant activity that ranged between 64% and 92% with cell viability of 97% on human dermal fibroblasts, indicating excellent biocompatibility of curcumin-loaded nanofibrous mats for wound healing application [143].

Liu and co-workers formulated PEG-silk fibroin electrospun nanofibrous membranes incorporated with curcumin. Curcumin release was stable for 350 h and the drug release increased with a decrease in the diameter of the fibers [144]. Zahiri et al. formulated PCL-gelatin electrospun nanofiber mats encapsulated with curcumin-loaded chitosan nanoparticles for applications in wound healing. The plain nanofibrous mats displayed a high tensile strength of 3.78 ± 0.17 MPa which decreased to 1.84 ± 0.12 MPa after the encapsulated of curcumin nanoparticles. The water contact angle studies of curcumin loaded nanofibrous mats exhibited hydrophilic nature with a contact angle of 48.9° ± 5.4°. The nanofibers possessed low degradation rate when compared to the plain mats and curcumin-loaded mats. The *in vivo* wound healing studies of PCL-gelatin electrospun nanofiber mats encapsulated with curcumin-loaded chitosan nanoparticles demonstrated high degrees of wound closure with an 82% wound closure on day 14 when compared to 73.4% wound closure for the plain nanofibrous mats [145].

### 5.5. Other Wound Dressing Materials

There are other bioactive wound dressing scaffolds which have been designed for the delivery of curcumin such as transdermal patches, wafers, foams, etc. Hegge and co-workers formulated alginate-based foams loaded with curcumin for wound treatment applications. The water absorption capability of the curcumin-loaded polymeric foams at model physiological pH was high revealing their suitability for highly exuding wounds. The *in vitro* drug release profile was slow and sustained. The drug-loaded foam displayed good phototoxic effect against *E. coli* [146].

Niranjan et al. formulated PVA-chitosan patches encapsulated with nano-curcumin for application in wound healing [147]. The swelling capability of the patches increased over time and are capable of absorbing wound exudates. The water vapor transmission rate (WVRT) value of the PVA-chitosan patches ranged between 2157–2299 g m^−2^ day ^−1^ which is an appropriate wound dressing scaffold. The *in vitro* drug release of curcumin was gradual in a controlled manner. The curcumin-loaded PVA-chitosan patches displayed significant high inhibition zones of 14 mm, 15 mm, 18 mm and 20 mm against *B. subtilis*, *S. aureus*, *E. coli* and *P. aeruginosa*, respectively, showing their good antibacterial effects against both Gram negative and Gram positive bacterial strains. Their cell viability of NIH3T3 cells after incubation was high after 72 h indicating the non-cytotoxic effect of patches. The wound healing evaluation *in vivo* showed that the wound areas treated with curcumin-loaded patches were completely closed with normal dermal environment with the appearance of hair on the 16th day when compared to the commercially available ointment treated wounds and untreated wounds [147].

Bulbake et al. formulated composite skin grafts from curcumin-loaded gelatin gel and cytomodulin-coupled porous PLGA microparticles. The composite skin grafts displayed a high interconnected microporous morphology. The fluid uptake was 40% with a drug release of 40% within 3 days followed by a prolonged and sustained drug release pattern for 7 days. The skin grafts’ tensile strength was high, elastic and flexible, indicating their capacity to stretch before breaking during the application of force. The *in vivo* wound healing assessments in the diabetic wound model demonstrated faster wound closure for wounds treated with composite skin grafts when compared to curcumin-loaded gelatin gel and control [148]. Shah and co-workers prepared polyurethane urea elastomers encapsulated with curcumin. The mechanical properties of all the designed elastomers were elastic with elongation at break ranging between 213.2% and 925.38%, Young’s moduli ranging between 2.18 and 23.33 MPa, and tensile strength ranging between 7.34 and 19.08 MPa. The *in vitro* antimicrobial analysis of curcumin-loaded elastomers exhibited superior antibacterial effects against *S. aureus* and *E. coli* [149].

Ternullo et al. designed soybean phosphatidylcholine-Polysorbate 20 deformable liposomes loaded with curcumin for skin wound healing. The *in vitro* cytotoxicity investigation demonstrated that both curcumin-loaded liposomes and plain liposomes were non-toxic when incubated with human skin fibroblasts (HFF cells) for 12 and 24 h, respectively. The *in vitro* antibacterial studies showed that all the curcumin-deformable liposomes inhibited *S. pyogenes* and *S. aureus* growth indicating their potential application on bacterial infected wounds [150]. Nguyen et al. synthesized amorphous nanoparticle complex of curcumin and oligochitosan for wound healing. They exhibited minimal cytotoxic on the HaCaT cells with cell viability of 89.6% and 99.4% at the high and low curcumin amounts, respectively. The *in vivo* wound healing analysis employing wounds on male mice (*Mus musculus var*) displayed that the nanoparticle complex of curcumin and oligochitosan exhibited superior wound healing activity with the wound closure of more than 90% after 7 days [151]. Chereddy et al. designed PLGA nanoparticles incorporated with curcumin. The drug release profile of curcumin was sustained for a period of 8 days. The curcumin-loaded polymeric nanoparticles showed an average particle size of 176.5 nm with an encapsulation efficiency of 89.2% and polydispersity index (PDI) of 0.105 with a negative zeta potential of −23.2 mV. The *in vivo* wound healing evaluation using RjHan: NMRI female mice full thickness excisional model with curcumin-loaded nanoparticles displayed significant healing on day 10 when compared to the plain nanoparticles (75% wound closure) and untreated wounds [152].

Karri et al. prepared collagen-alginate scaffolds encapsulated with synthesized curcumin-incorporated chitosan nanoparticles for diabetic wound treatment. The average particle size of the scaffold was 196.4 nm with a positive surface charge of 30 mV. The pore sizes ranged between 50 and 250 µm. The swelling capacity ranged between 820–910%, depending on cross-linking within the polymers. The *in vitro* cytotoxicity of the polymeric scaffolds showed low cytotoxic effects on 3T3-L1 fibroblasts. The *in vivo* wound closure studies using diabetic Wister mice showed that the average percentage of wound contraction was significantly higher by 98.1% for the curcumin-nanoparticle loaded scaffolds when compared to those of the sterile gauze (control) (44.6%) and plain scaffolds (61.6%) [153]. Rezaii and co-workers prepared collagen-chitosan scaffolds incorporated with curcumin nanoparticles for cutaneous wound healing. The wound healing analysis *in vivo* utilizing male Wistar mice showed a significant contraction of the wound area treated with curcumin-nanoparticle loaded scaffolds when compared to the plain scaffold, indicating that the incorporation of curcumin nanoparticles into the scaffolds resulted in improved wound closure [154]. Venkatasubbu and Anusuya formulated curcumin nanocomposite loaded with silver nanoparticles and prepared from poly vinyl alcohol. It was prepared for wound treatment. The DLS analysis of the nanocomposite showed mean hydrodynamic diameter ranging between 290 and 350 nm. 100% of the loaded curcumin was released over a period of 25 h. The release mechanism of curcumin from the coated cotton cloth was sustained with a controlled initial burst release of curcumin. The antimicrobial assessments *in vitro* revealed their broad spectrum bacterial growth inhibitory effect against *P. vulgaris*, *S. epidermis*, *E. aerogenes*, *K. pneumonia*, and *P. mendocina* indicating that these scaffolds can be beneficial for microbial infected wounds [155].

## 6. Conclusions

Most polymer-based wound dressings exhibit unique properties that are suitable for wound healing process such as their ability to offer a moist environment for an accelerated wound healing mechanism, good biodegradability for tissue regeneration, gaseous permeability to allow the diffusion of CO_2_, O_2_, and water vapor and high porosity useful for the transportation of nutrients and liquids. Despite the aforementioned interesting features, most wound dressings do not protect the wound from bacterial invasion, exhibiting poor antioxidant and anti-inflammatory activity. The addition of curcumin into prepared wound dressings has been reported to result in improved mechanical properties (such as high flexibility, elongation at break, tensile strength, and elasticity), enhanced absorption capacity, prevention of bacterial invasion and treatment of bacterial infected wounds. The rate of wound healing was accelerated when wound dressings loaded with curcumin was used. Furthermore, the combination of curcumin with metal-based nanoparticles (such as silver and titanium) resulted in synergistic antibacterial effects. The release profile of curcumin from the designed wound dressings was controlled and sustained with an initial burst effect suitable for bacterially infected wounds. Most of the described studies are at the preclinical phase and their outcomes are promising. However, there is a need for these wound dressings to reach clinical trials.
